# Physical Properties of E143 Food Dye as a New Organic Semiconductor Nanomaterial

**DOI:** 10.3390/nano13131974

**Published:** 2023-06-29

**Authors:** Mohammed Alyami, Satam Alotibi

**Affiliations:** Department of Physics, College of Science and Humanities in Al-Kharj, Prince Sattam Bin Abdulaziz University, Al-Kharj 11942, Saudi Arabia; sf.alotibi@psau.edu.sa

**Keywords:** E143 food dye, organic semiconductors, interband transitions, electrical conductivity, dielectric relaxations

## Abstract

Organic semiconductors (OSCs) have attracted considerable attention for many promising applications, such as organic light-emitting diodes (OLEDs), organic field-effect transistors (OFETs), and organic photovoltaics (OPVs). The present work introduced E143 food dye as a new nanostructured organic semiconductor that has several advantages, such as low cost, easy fabrication, biocompatibility, and unique physical properties. The material was characterized using a transmission electron microscope (TEM), Fourier transform infrared (FT-IR) spectroscopy, thermogravimetric analysis (TGA), and optical absorption spectroscopy. The study of X-ray diffraction (XRD) showed that E143 dye has a monoclinic polycrystalline structure. Electrical and dielectric properties were performed by impedance spectroscopy at frequencies (20 Hz–1 MHz) in the temperature range (303–473 K). The values of interband transitions and activation energy recommended the application of E143 dye as a new organic semiconductor material with promising stability, especially in the range of hot climates such as KSA.

## 1. Introduction

**Figure 1:**
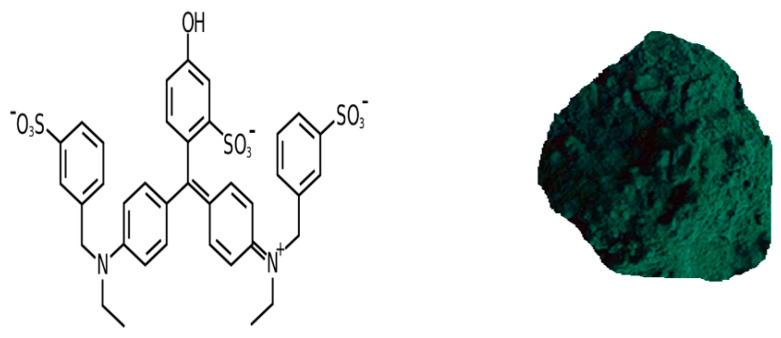
The chemical structure and physical form of E143 food dye (C_37_H_34_N_2_Na_2_O_10_S_3_).

Organic semiconductors (OSCs) are compounds with a complex aromatic molecular structure and fixed color for many potential applications, such as photovoltaics (PVs), electronics, photosensors, and nonlinear optics [1,2,3]. Unlike conventional semiconductors, OSCs are solid crystalline materials whose building blocks are π-bonded molecules of carbon and hydrogen atoms and sometimes other elements such as nitrogen, sulfur, and oxygen. Numerous commercial products employing organic dyes as OSCs have appeared in the market in different fields, such as down-shifting PV coatings [4], flat-screen displays [5,6], and many industrial processes [7]. A great interest has been devoted to organic dyes to understand the physical processes that can occur in the devices based on these materials [8]. Charge transport is one of the important processes where charge carriers (electrons or holes) move through organic dyes or between organic dyes and other materials, such as electrodes or semiconductors [9,10]. This process can affect the performance and efficiency of devices based on organic dyes, such as dye-sensitized solar cells (DSSCs) [11], organic field-effect transistors (OFETs) [12], and organic light-emitting diodes (OLEDs) [13]. Organic dyes are usually used as active OSCs for OLED used for digital displays in several electronic products [14]. Some examples of OSCs for OLEDs are organometallic chelates such as Alq3, fluorescent and phosphorescent dyes, and conjugated dendrimers. These dyes can be incorporated into different layers of OLEDs, such as the hole injection layer (HIL), the hole transport layer (HTL), and the emissive layer (EML) [15]. Recent studies on the electrical conductivity of natural organic dyes have been performed to understand their potential applications in electronic devices [16,17]. This study has important features depending on the dye molecular structure, crystallinity, and temperature by which the conduction mechanisms can be revealed [18,19]. The physical properties of organic dyes are currently an important motivation in many fields of applied research [20]. The development of nanostructured organic dyes by aromatic rings, conjugated systems, heterocycles, or metal complexes has opened the way to enhance the processing and physical properties for different applications [21]. E143 (C_37_H_34_N_2_Na_2_O_10_S_3_) is a turquoise triarylmethane food dye; this dye is a derivative from hydrocarbon, triphenylmethane, tertiary alcohol, and triphenylcarbinol groups [22]. Figure 1 illustrates the chemical structure of E143 dye, it is structurally like azo dyes substituted with a phenolic hydroxyl and two sulfonic acid groups. However, the complex aromatic molecular structure of this dye make it more stable and difficult to be biodegraded [23]. E143 food dye is usually supplied as a crystalline solid in the form of dark green powder or granules with a metallic luster, and its brilliant color is less likely to fade. E143 food dye is used in many potential applications such as food industries [24,25,26], medical applications [27,28,29], and waste water treatment [30]. It is also used as a quantitative stain for histones at alkaline PH after acid extraction of DNA as well as protein stain in electrophoresis [31]. It is considered a safe stain for tumor detection as it forms stable-colored complexes with histones [32]. The present work aims to introduce a new line of the applications of E143 food dye as an organic semiconductor material, this is achieved by studying the structure, interband transition, electrical conductivity, and the dielectric properties to check whether it can be applied to organic semiconductor-based devices.

## 2. Experimental Techniques

E143 food dye (certified by the Biological Stain Commission) was obtained from Merck, Germany, with the molecular formula C_37_H_34_N_2_Na_2_O_10_S_3_ and a molecular weight of 808.85 gm; this dye is usually supplied as a crystalline solid and stable for two years at room temperature. TEM measurement was carried out using a transmission electron microscope (JEOL JEM-1400, Tokyo, Japan). E143 dye powder was dissolved in ethanol with a concentration of 10^−4^ wt% and then loaded on a carbon-coated copper grid. The grid was masked by a protective film to avoid material damage by an electron beam. Other precautions were taken, such as fast scanning and reduced beam current [33]. A Fourier transform (FT-IR) spectrum was recorded by (NICOLET 6700 FT-IR, Waltham, MA, USA) in the wavenumber range of 4000–400 cm^−1^. TGA for E143 was performed in the temperature range 40–900 °C in a nitrogen atmosphere at a heating rate of 10 °C/min using a thermogravimetric analyzer. X-ray diffraction (XRD) patterns were recorded by x-ray diffractometer (SHIMADZUXRD-6100,Tokyo, Japan), using CuKα (λ = 1.5406 Å) radiation over 2θ, ranging from 5 to 90° with a scan speed 2 deg/min. The optical absorption spectrum 10^−4^ wt% E143 dye ethanol solution was recorded at room temperature in the wavelength range (300–1100 nm) using a spectrophotometer type (JASCO, V-770, UV–VIS-NIR, Tokyo, Japan). Direct (DC) and alternating current (AC) was measured; the dye was compacted in the form of circular discs of area 0.0065 m^2^ and thickness (1.6 mm) and heated in a shielded homemade furnace in the temperature range (298–383 K). DC electrical conductivity was determined from the resistance measured directly using a digital electrometer (Keithley 616, Ohio, USA). The measurements of AC electrical conductivity and dielectric properties were carried out in the frequency range of 100 Hz–1 MHz using an impedance meter (Wayne Kerr-6440B RLC, MA, USA), with accuracy (±0.005%).

## 3. Results and Discussion

### 3.1. TEM Characterization

**Figure 2:**
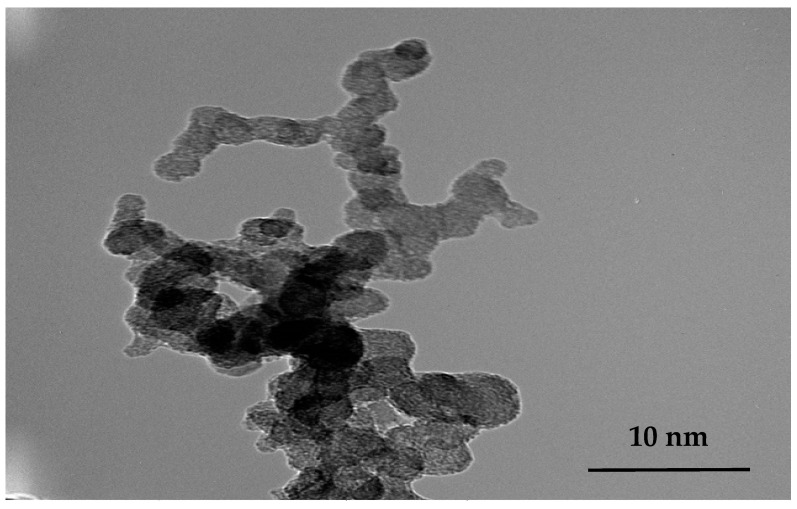
TEM image of nanosized E143 food dye (C_37_H_34_N_2_Na_2_O_10_S_3_).

Figure 2 shows a TEM image of E143 food dye (C_37_H_34_N_2_Na_2_O_10_S_3_) dissolved in ethanol, and it is clear from the scale bar that organic molecules have a cluster form of nanosized structure. This property enables a structural diversity for easily tunable photoelectronic properties via low-temperature chemical processing and adjustable band gap of OSC applications [3,34]. It was reported that promising subwavelength structures could manipulate the amplitude, phase, frequency, and polarization state of light for various applications, such as nonlinear optics, surface photocatalysis, surface-enhanced spectroscopy, and photodetection [35,36].

### 3.2. FT-IR Spectroscopy

**Figure 3:**
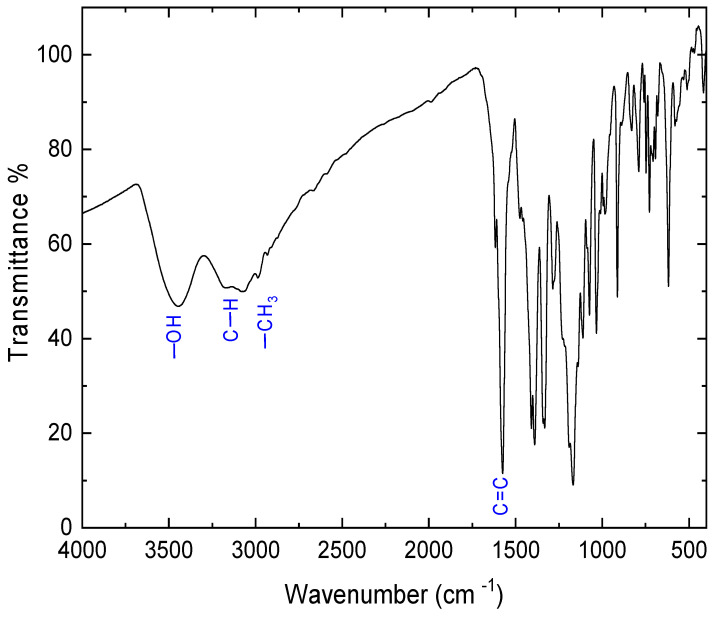
FT-IR of E143 food dye (C_37_H_34_N_2_Na_2_O_10_S_3_).

FT-IR transmittance spectra of E143 food dye (C_37_H_34_N_2_Na_2_O_10_S_3_) in powder form are demonstrated in Figure 3. The important characteristic bands, along with the main functional groups, were determined. The broadband observed at high energy, located around 3443.2 cm^−1^, can be assigned to the hydroxyl group (–OH). The vibrational modes recorded at 3181.8 cm^−1^ can be assigned to the asymmetric stretching vibration of the aliphatic C–H bond, whereas the band that appeared at 3064 cm^−1^ may be raised from the vibrational resonance of the –CH_3_ bond. In addition, a very strong vibration mode that appeared at 1575 cm^−1^ can be due to the aromatic ring C=C [37]. Generally, the functional groups depicted by FT-IR spectra are well correlated to the chemical structure of the E143 compound.

### 3.3. Thermogravimetric Analysis

**Figure 4:**
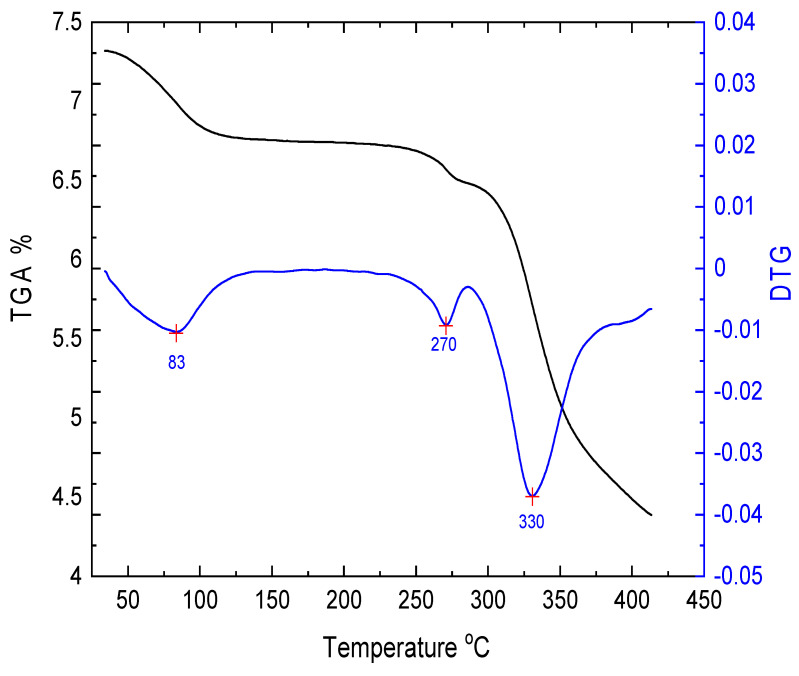
TGA and DTGA of E143 food dye (C_37_H_34_N_2_Na_2_O_10_S_3_).

Thermogravimetric (TGA) analysis is an important tool for examining the thermal stability of different materials, especially organic materials, which usually have low melting points due to their weak intermolecular forces. Figure 4 shows the TGA curve and the derivative (DTGA) thermographs for E143 food dye (C_37_H_34_N_2_Na_2_O_10_S_3_) processed in powder form. It was observed that TG curves exhibit three weight loss steps that occurred in the range (30–100 °C), (100–250 °C), and (250–400 °C), respectively. In the first step, the initial weight loss is usually attributed to the evaporation of superficial humidity and traces of volatile constituents that are present in the dye. Subsequently, a further slight weight loss occurred in the second step (100–250 °C), showing the thermal stability of the dye in this temperature range. Finally, in the last step (260–400 °C), a large percentage of the total mass was lost due to the decomposition of the dye molecules as a result of thermal degradation [38]. The decomposition suggests that the TGA curve during heating can be clearly observed in the diagram of DTGA. An inspection of the DTGA curve showed the first phase transition at 82 °C followed by decomposition temperatures at 270 °C and the melting point at 330 °C, respectively. These values are high due to the existence of many carbon atoms compared to low molecular-weight organic dyes. This study endorses the promising thermal stability of E143 and recommends its use in electrical and solar energy applications in a wide temperature range.

### 3.4. X-ray Diffraction

**Figure 5:**
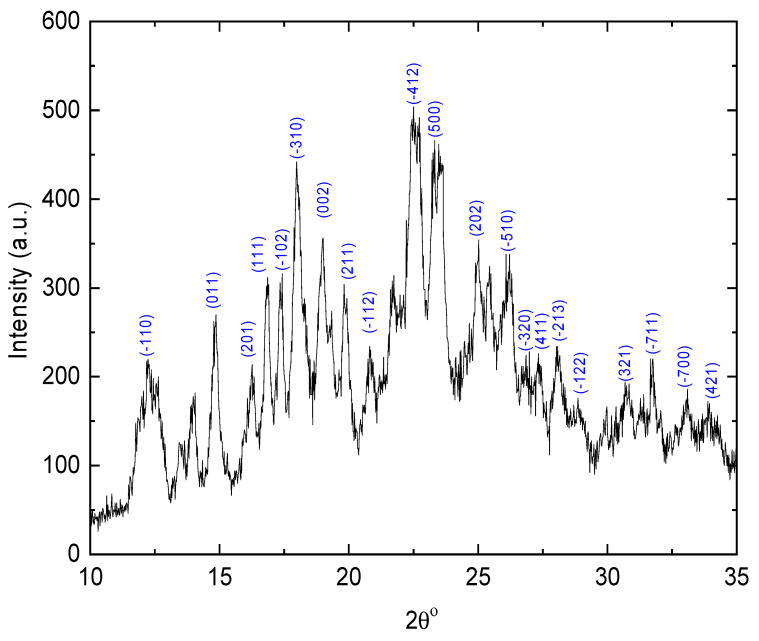
XRD pattern of E143 food dye (C_37_H_34_N_2_Na_2_O_10_S_3_).

XRD spectra of E143 food dye (C_37_H_34_N_2_Na_2_O_10_S_3_) in powder form were recorded in the 2θ range from 5 to 90°; the characteristic peaks were found in the range of 10–35°, as shown in Figure 5. The maximum intensity is observed at 22.59° for the plane −412, while the sharpest peaks appeared at 14.82°, 16.84° and 17.30°, and 22.54° corresponding to the planes (011), (111), and (−102), respectively. All the diffraction peaks in the powder spectra were indexed, and Miller indices *(hkl*) were determined with the aid of the CRYSFIRE computer program. The refinement of the obtained lattice parameters via the experimental and the calculated 2θ° was determined using the CHEKCELL program [39]. The obtained *hkl*, observed and calculated 2θ with the difference between these values, are listed in Table 1. It is observed that E143 has a polycrystalline nature with a monoclinic crystal structure; the calculated lattice parameters are *a* = 11.5442 Å, *b* = 7.7879 Å, *c* = 10.5705 Å, α=γ=90°,β=118.21°).

### 3.5. Optical Absorption

**Figure 6:**
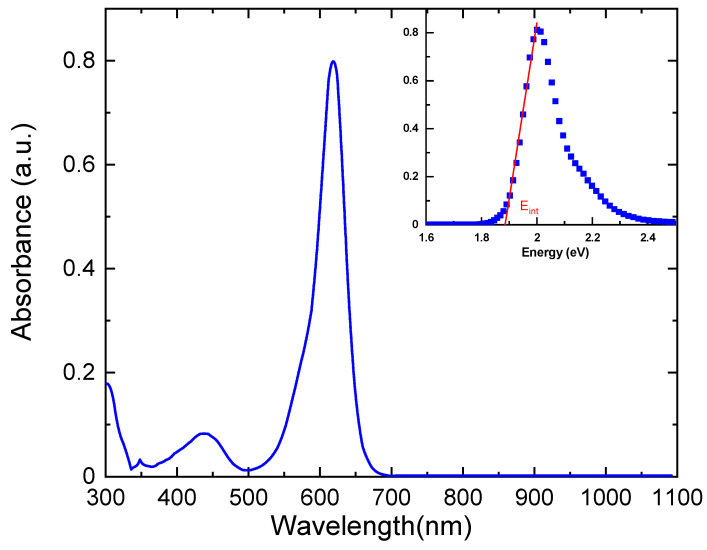
Optical absorption spectra of E143 food dye (C_37_H_34_N_2_Na_2_O_10_S_3_) dissolved in ethanol with concentration 10^−3^ wt%. The interband band transition, E_int_, is indicated inset.

Figure 6 illustrates the room temperature optical absorption spectrum of E143 food dye (C_37_H_34_N_2_Na_2_O_10_S_3_) dissolved in ethanol with a concentration of 10^−4^ wt%, and the material exhibits a wide UV–vis absorption spectrum that covers the wavelength range from 350 to 700 nm. The spectrum shows two major absorption bands; the first band can be due to transitions from the ground state to the second excited state (S_0_→S_2_) around 437 nm, which is called the B band or the Soret band [40]. The second absorption band is due to the transition from the ground state to the first excited state (S_0_→S_1_) around 618 nm, which is called the Q band [40]. Both the B and Q bands are due to π-π* transitions and can be understood by Gouterman four orbital model by looking at the far frontier orbitals (HOMO and LOMO) [41]. The B band is usually located in the ultraviolet region, but it can shift to the visible region depending on the structure and environment of the molecule [42]. For example, metal complexes of phthalocyanines and porphyrins can have B bands in the visible region due to the interaction between the metal and the ring system [43].

Interband transition is a process in which a photon can excite an electron from an occupied state in the valence band to an unoccupied state in the conduction band of a material, and this process involves the absorption of the photon and the creation of a hole in the valence band [42]. Interband transitions can be either direct or indirect, depending on whether the crystal momentum of the electron is conserved or not. The absorption spectrum was replotted inset Figure 6 as a function of photon energy to determine the interband energy transition, E_int_, from HOMO to LUMO from the intercept at the energy axis [44]. It is noted that the value of E_int_ is 1.885 eV lies in the range of semiconducting materials (0.1–3 eV) [17]. This feature means that charge carriers can contribute to the electrical conductivity due to the creation of an electron–hole upon photon absorption by E143 dye molecules [45]. The efficiency and mechanism of interband transitions depend on several factors, such as the type of band gap (direct or indirect), the presence of impurity states or defects, the crystal structure and composition of the semiconductor, and the temperature [46].

### 3.6. DC Conductivity

**Figure 7:**
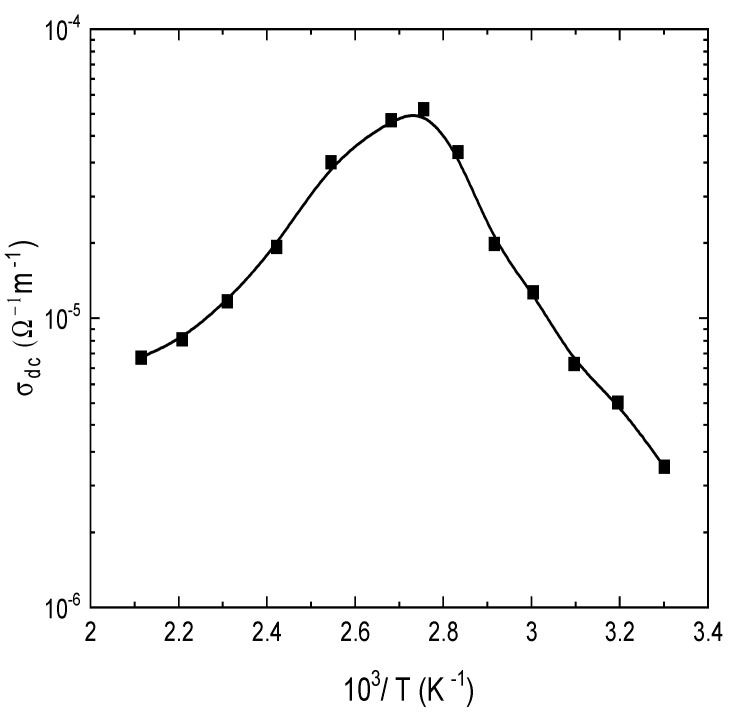
Temperature dependence of DC electrical conductivity, σ*_dc_*, of E143 food dye (C_37_H_34_N_2_Na_2_O_10_S_3_).

The temperature effect on the DC electrical conductivity, *σ_dc_*, was studied for circular discs made of E143 food dye (C_37_H_34_N_2_Na_2_O_10_S_3_) at different temperatures in the range (303–473 K) and plotted as shown in Figure 7. It was observed that *σ_dc_* is thermally activated up to a peak value located at 353 K and then decreased at higher temperatures. This behavior suggested the existence of two different conduction mechanisms, the first operates in the low-temperature range (303–353 K), and the second occurs in the range (353–473 K). The behavior of DC conductivity can be described using the Arrhenius equation [16],

(1) $$\sigma_{dc}=\sigma_{{}^{\circ}}\exp\left(-\frac{{∆E}_{\sigma }}{k_{B}T}\right)$$

where ∆*E_σ_* is the activation energy for the electrical conduction, *T* is the absolute temperature; *k_B_* is Boltzmann’s constant, and *σ*_o_ is the pre-exponential factor depending on the density of states and the charge carrier mobility. The calculated values of the activation energy of the two regions ∆*E_σ_*_1_ and ∆*E_σ_*_2_ were 0.10 eV and 0.24 eV, respectively. At region (1), the electrical conductivity of the dye is increased by increasing temperature, with similar behavior as observed in semiconductors. Additionally, the calculated activation energy confirms that the dye is an organic semiconductor [47]. This behavior implies that as the temperature is increased, the charge carriers gain higher mobility and overcome the activation energy barrier and contribute to the electrical conduction. The observed increase in the conduction can be associated with the increased mobility of electrons of the organic compounds [48] and reveals the semiconducting behavior of E143 dye. At higher *C*temperatures above 353 K, the values of *σ_dc_* are decreased by increasing temperature due to the phase changes as verified by TGA studies. This behavior may be ascribed to the lattice disorder caused by thermal vibrations, which cause random motions of charge carriers from one site to another and hinder the conduction process.

### 3.7. AC Conductivity

**Figure 8:**
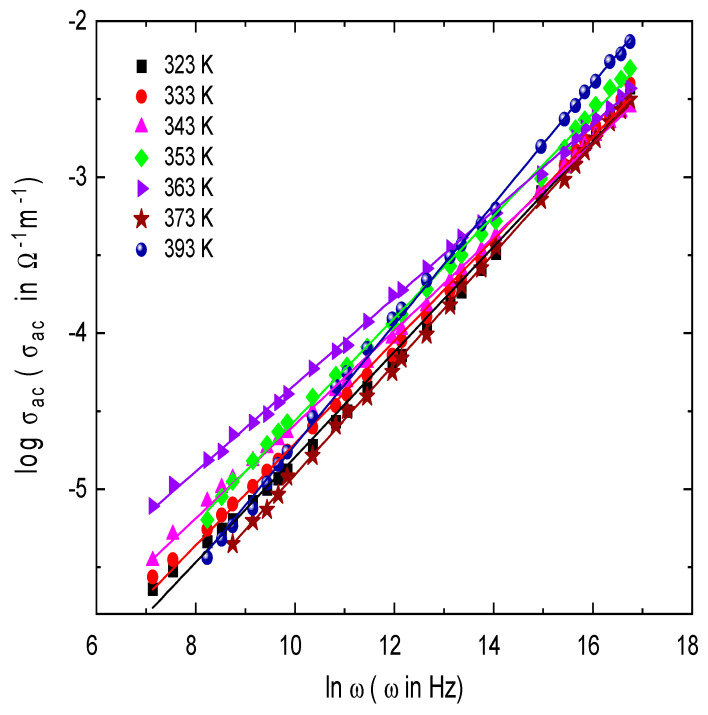
Frequency dependence of AC conductivity, σ_ac_, for E143 food dye (C_37_H_34_N_2_Na_2_O_10_S_3_) at different temperatures.

**Figure 9:**
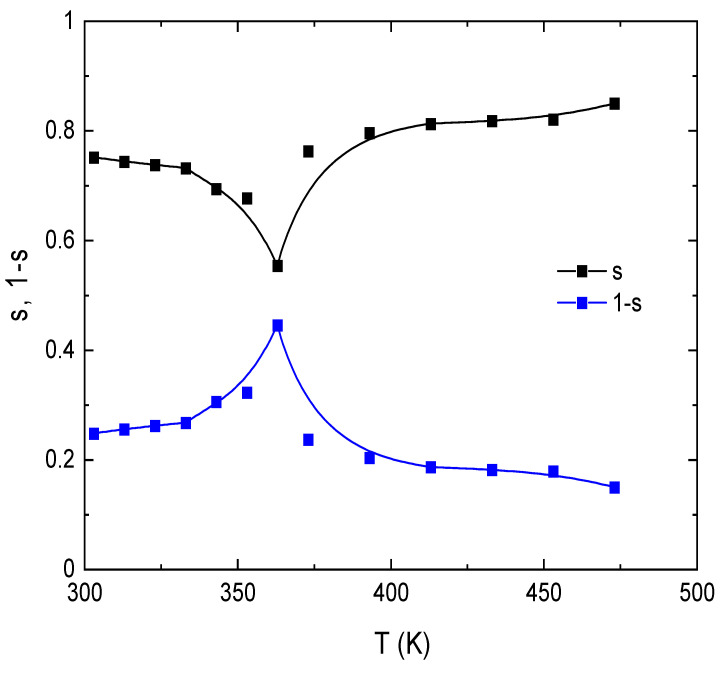
Temperature dependence of the frequency exponent, s, and 1-s, for E143 food dye (C_37_H_34_N_2_Na_2_O_10_S_3_).

The frequency dependent on AC electrical conductivity for semiconductor materials is an important study to explain the conduction mechanism. Understanding the conduction process gives valuable information about the defect levels present in the system and also helps to differentiate between localized and free band conduction. Regarding the localized conduction, *σ_ac_* increases with frequency, while *σ_ac_* decreases with frequency in the free band conduction. The total conductivity, *σ_total_* at angular frequency ω, can be written as [18,49],

*σ_total_ = σ_dc_ + σ_ac_* (2)

where *σ_dc_* and *σ_ac_* are the DC and AC conductivities; it is assumed that *σ_dc_* represents *σ_ac_* at zero frequency. In many semiconductors and insulators, the frequency dependence of *σ_ac_* can be expressed by [18,49],

(3) $$\sigma_{ac}\left(\omega \right)=A\omega^{s}$$

where *A* is a temperature-dependent constant; ω is the angular frequency; and *s* is the frequency exponent, which controls the type of the conduction mechanism and is generally smaller than or equal to unity. Figure 8 shows the frequency dependence of *σ_ac_* in the frequency range from 30 Hz to 1 MHz and a temperature range of 303–473 K for circular discs made of E143 food dye (C_37_H_34_N_2_Na_2_O_10_S_3_). It is noted that *σ_ac_* increases with increasing frequency; this behavior is ascribed to the electron jumping (hopping and/or tunneling) between electron donor atoms and empty sites localized in the band gap [50]. The values of the frequency exponent *s* were directly obtained by calculating the slopes of Figure 8 at the studied temperature range. The temperature dependence of *s* is shown in Figure 9; the values are decreased with increasing temperature to 353 K and then increased.

Many models have been postulated in order to explain the AC conduction in semiconductors based on the frequency and temperature dependence of the frequency exponent *s*. Quantum Mechanical Tunneling (QMT) [51] and Correlated Barrier Hopping (CBH) are the most well-known models used for this issue [19,52]. Regarding these models, three types of charge carriers are distinguished: electrons, small polarons, and large polarons. In the CBH model, the charge carriers jump over the potential barrier between two defect states over the potential barrier separating them. In the temperature range T < 383 K, the exponent *s* is decreased with temperature obeying the CBH model according to [53],

1-*s* = 6*k_B_T/W_m_* (4)

where *W_m_* is the carrier in the localized states. The calculated value of *W_m_* was 0.34 eV, as obtained by calculating the slope of the 1-*s* versus *T* relation plotted in Figure 9. It is obviously noted from the extrapolation of the 1-*s* plot that the value of s approaches one as *T* tends to zero K, so the CBH model is the dominant conduction mechanism at *T* < 353 K. On the other hand, at temperature values *T* > 353 K, the value of *s* increases by raising the temperature signifying the typical behavior of the QMT model. In the non-overlapping small polaron tunneling (NSPT) model, *s* is only temperature dependent as it slightly increases with increasing temperature. Thus NSPT model cannot be applied to determine the conduction mechanisms for the obtained results. According to the overlapping large polaron tunneling (OLPT) model, the value of *s* is decreased by increasing temperature until reaching a minimum value at a given temperature and then continues to increase by increasing temperature [54]. This model discussed *σ_ac_* predicted from the QMT model in which tunneling of polarons is the dominant mechanism along with considerable overlap of polaron distortion clouds. For large polarons, the spatial extent is large compared with the inter-atomic spacing; thus, the potential wells of neighboring sites are overlapped. As a result of the dominant Coulombic interaction, the polaron hopping energy is reduced. According to the OLPT model, the frequency exponent “*s*” can be evaluated by [55],

(5) $$s=1-\frac{8\alpha R_{\omega }+6W_{H0}r_{p}/R_{\omega }k_{B}T}{{\left(2\alpha R_{\omega }+\frac{6W_{H0}r_{p}}{R_{\omega }k_{B}T}\right)}^{2}}$$

where *R_ω_* is the hopping length, *r_p_* is the polaron radius, and *W_H_*_0_ is the hopping polaron energy assumed to be constant for all sites given by [55],

*W_H_*_0_ = *e^2^*/4*ε_p_ r_p_* (6)

**Figure 10:**
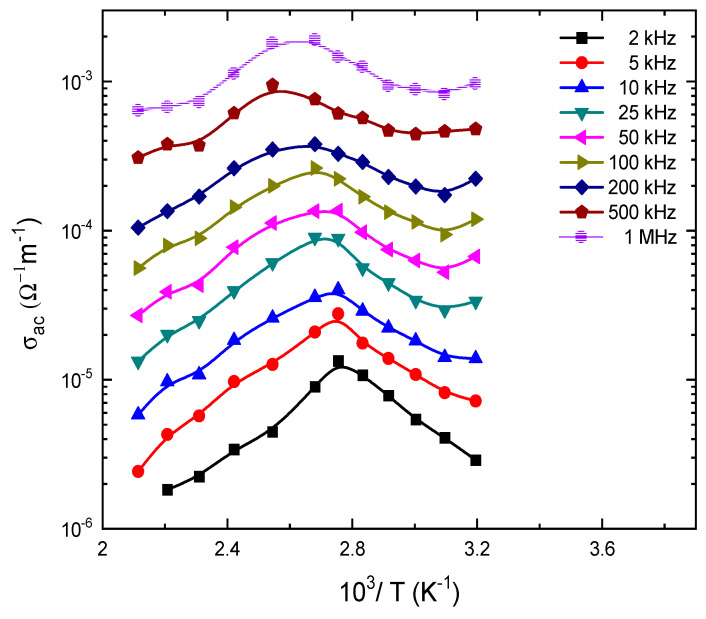
The temperature dependence of AC conductivity, *σ_ac_*, for E143 food dye (C_37_H_34_N_2_Na_2_O_10_S_3_).

Thus, the OLPT model suggests that the frequency exponent *s* should be temperature and frequency dependent and decreases from unity by increasing temperature. For large values of 2*αr_p_*, *s* is decreased, tending to the value predicted by the QMT model. On the other hand, for the small values of 2*αr_p_*, *s* exhibits a minimum value at a certain temperature and then increase by increasing temperature in a similar way to the case of small-polarons according to the QMT model. OLPT conduction mechanism suggests the application of E143 food dye as a hole injection layer (HIL) for OLED applications since large polarons can tunnel through the potential barriers created by overlapping lattice distortions [56,57]. This can be ascribed to the fact that large polarons can affect the charge injection and transport in OLEDs by modifying the energy barriers and tunneling rates at the interfaces between different organic layers [58,59]. The temperature dependence of *σ_ac_*(*ω*) is shown in Figure 10 at different frequencies. It is clear that *σ_ac_*(*ω*) is thermally activated up to a peak value at a certain temperature and then continues to decrease by increasing temperature in agreement with the behavior observed for *σ_dc_* results. This peak is shifted to higher temperatures by increasing the frequency of the applied field due to the enhancement of carrier jumping. This can be ascribed to the increase in carrier mobility according to the thermal activation jumping model.

**Figure 11:**
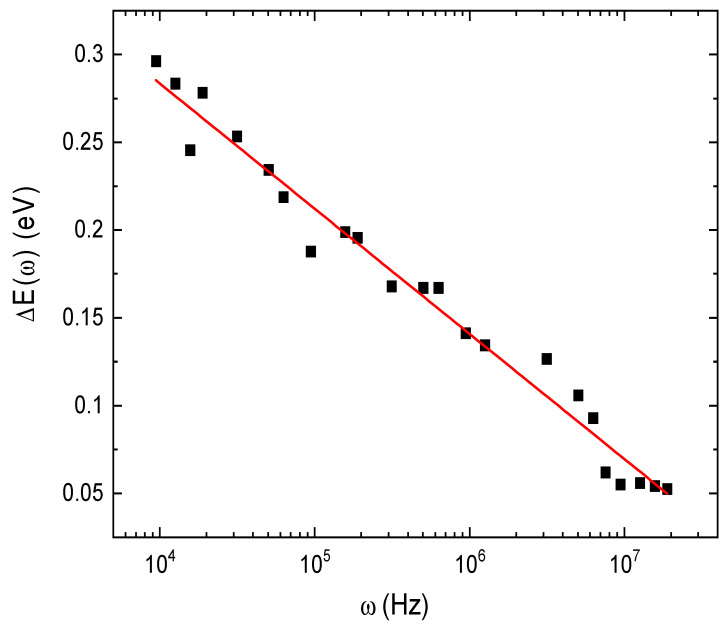
Linear Fitting of the frequency dependence of AC activation energy for E143 food dye (C_37_H_34_N_2_Na_2_O_10_S_3_).

The temperature dependence of *σ_ac_*(*ω*) is controlled by the thermal activation process according to the Arrhenius equation [60],

(7) $$\sigma_{ac}\left(\omega \right)=\sigma_{o}\exp\left(-\frac{∆E\left(\omega \right)}{k_{B}T}\right)$$

where ∆*E*(*ω*) is the activation energy for the electrical conduction. The AC activation energy was calculated for all the investigated frequencies; the frequency dependence of ∆*E*(*ω*) for E143 dye is shown in Figure 11. It is clearly noted that ∆*E*(*ω*) decreases by increasing frequency; such a decrease can be due to the contribution of the applied frequency to the conduction mechanism. This result suggests that hopping conduction is the dominant mechanism. It was observed that the values of ∆*E*(*ω*) are decreased by increasing frequency; this can be due to the fact that the increase in the frequency improves the electronic jumps between the localized states.

### 3.8. Dielectric Properties

**Figure 12:**
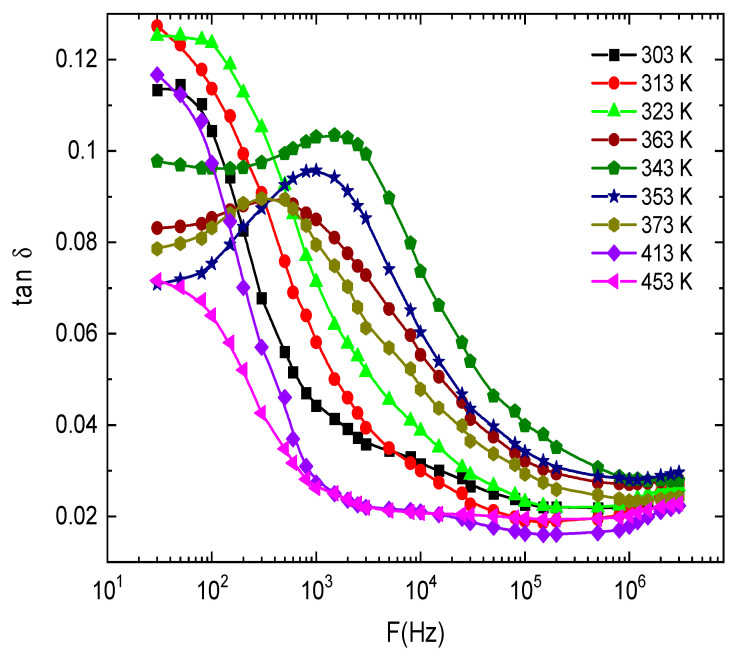
The frequency dependence of *tanδ* at different temperatures for E143 food dye (C_37_H_34_N_2_Na_2_O_10_S_3_).

**Figure 13:**
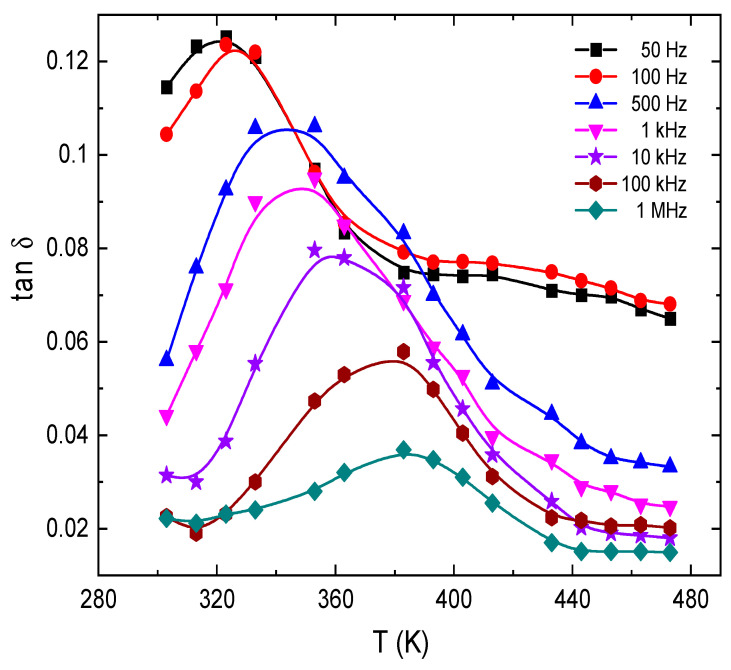
Temperature dependence of *tanδ* for E143 (C_37_H_34_N_2_Na_2_O_10_S_3_) dye.

Figure 12 shows the effect of frequency on the loss factor tan δ for E143 food dye (C_37_H_34_N_2_Na_2_O_10_S_3_). It is noted that the values of *tanδ* are high at low frequencies; this can be due to the Ionian conduction loss besides the loss of the electronic polarization. However, in the high-frequency region, there is one mechanism that controls the process, the oscillation in the Ionian dielectric loss [61]. The temperature dependence of tanδ is illustrated in Figure 13 for E143 food dye (C_37_H_34_N_2_Na_2_O_10_S_3_) at selected frequencies. It was noted that all the plots have one broad maximum, which is attenuated and shifted towards higher frequencies by increasing temperature. This attenuation can be due to the phonon dipole interaction, which leads to a decrease in the energy transported to the dielectric medium. This behavior is characteristic of the dielectric relaxations caused by the localized movement of the segments in the dye molecules.

**Figure 14:**
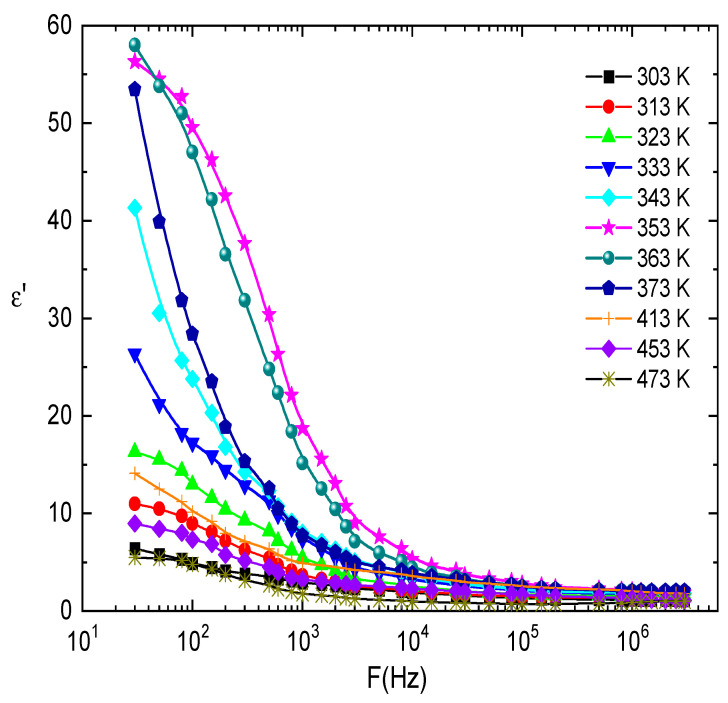
The frequency dependence of the dielectric constant, *ε*′, at different temperatures for E143 (C_37_H_34_N_2_Na_2_O_10_S_3_) dye.

**Figure 15:**
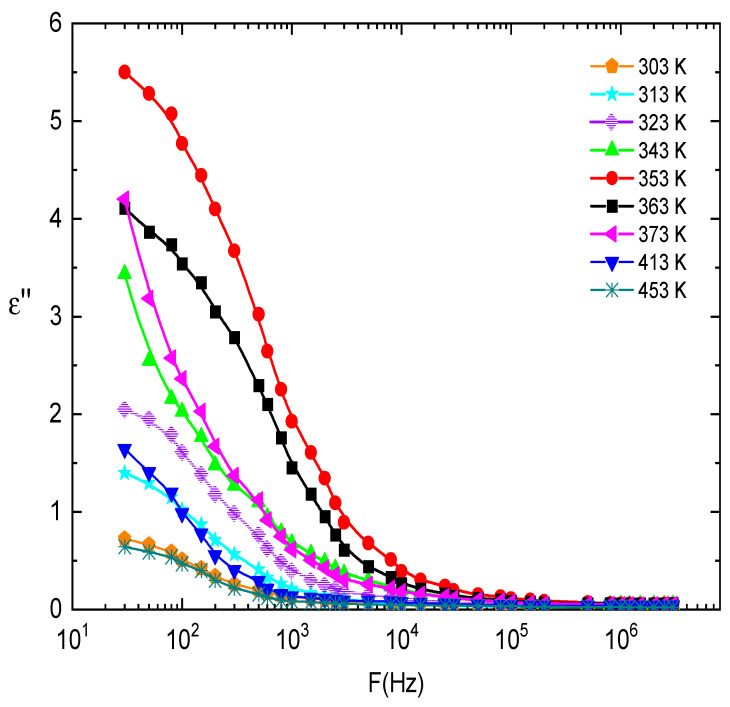
The frequency dependence of the dielectric loss, $\varepsilon^{\prime \prime }$, at different temperatures for E143 food dye (C_37_H_34_N_2_Na_2_O_10_S_3_).

The study of dielectric relaxation is very important to understand the origin of dielectric losses as a potential tool for the determination of the structure and defects existing in solids. The complex dielectric function for the investigated organic dye is expressed by the Debye equation [62],

(8) $$\varepsilon^{*}\left(\omega \right)=\varepsilon^{\prime }\left(\omega \right)+i\varepsilon^{\prime \prime }\left(\omega \right)$$

where (*ω*) and (*ω*) are the real and imaginary parts of the dielectric constant, respectively. The values of (*ω*) and (*ω*) were determined from the measurements of the capacitance and loss tangent *tanδ* under different temperatures and frequencies. Figure 14 and Figure 15 show the frequency dependence of (*ω*) and (*ω*) for E143 food dye (C_37_H_34_N_2_Na_2_O_10_S_3_) recorded at different temperatures. It was observed that (*ω*) is decreased by increasing frequency; this behavior can be explained by the dielectric polarization mechanisms of materials, which usually occur as interfacial, dipolar, ionic, and electronic polarization [63]. Electronic and ionic polarizability is active in the high-frequency range, while the other two mechanisms occur at low frequencies [64]. The dielectric molecules consist of negative charges centered around the positive charges located at the center of the molecule. When these molecules are subjected to an external electric field, the positive charges are biased toward the field, while the negative charges are biased to the opposite direction. As a result, the positive charge center is no longer applicable with the negatively charged center but separated by a small distance, causing the emergence of electrical dipoles, then the molecules become polarized and gain dipole moment. The effect of interfacial polarization appears at low frequencies and vanishes at higher frequencies, while the electronic and ionic polarizations come to be pronounced [65]. The decrease in (*ω*) with frequency may be ascribed to the fact that the migration of dipoles is the main source of the dielectric loss [66]. Accordingly, low and moderate frequencies are categorized by high values of (*ω*) due to the occurrence of carrier jumping in addition to polarization. On the other hand, at higher frequency values, the dipole vibrations may be the only source of dielectric loss, so (*ω*) decreases at high frequencies [66]. The obtained results of (*ω*) can be explained on the basis of the theory of hopping of the charge carriers over the potential barrier between defect sites, as suggested by Elliot [67].

**Figure 16:**
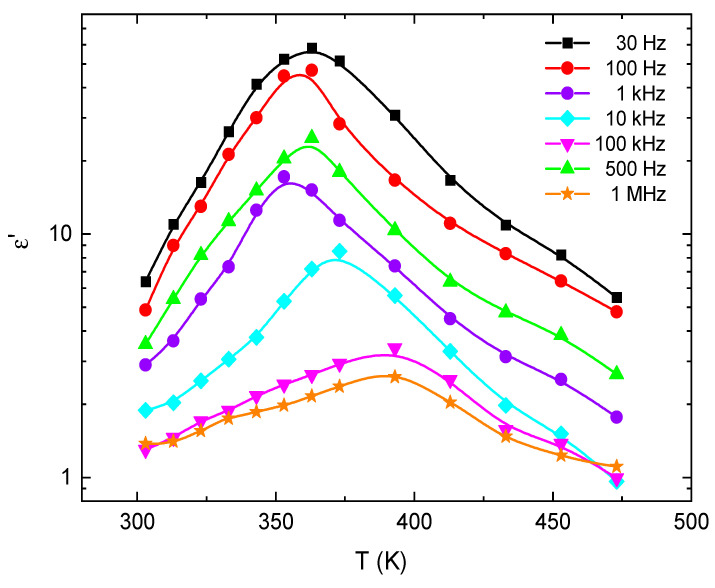
The variation in *ε*′ with the temperature at different frequencies for E143 food dye (C_37_H_34_N_2_Na_2_O_10_S_3_).

**Figure 17:**
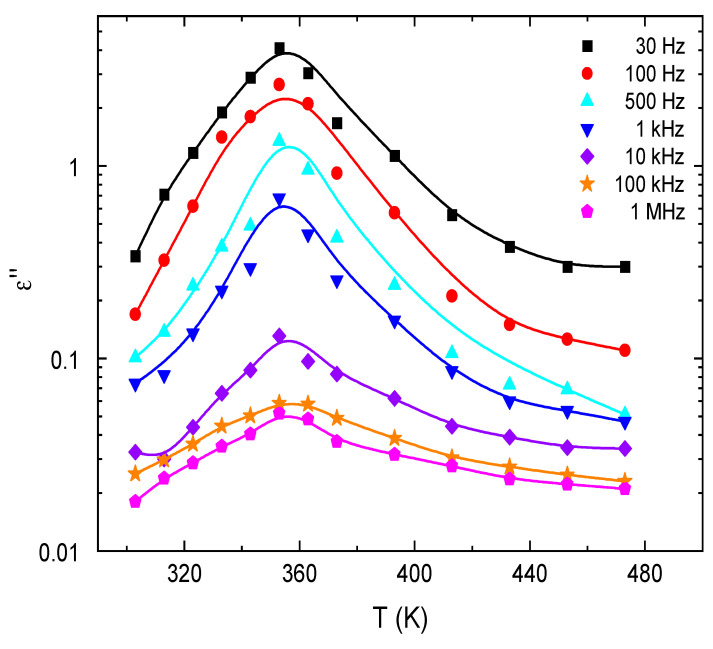
The variation in *ε*″ with the temperature at different frequencies for E143 food dye (C_37_H_34_N_2_Na_2_O_10_S_3_).

The temperature dependence of (*ω*) and (*ω*) for E143 food dye (C_37_H_34_N_2_Na_2_O_10_S_3_) was studied at different frequencies and plotted in Figure 16 and Figure 17. The increase in (*ω*) by increasing temperature can be due to the increase in polarization caused by the thermal motion of electrons [68]. Since the dipoles cannot orient themselves at low temperatures, their orientation is strongly facilitated by heating the material, and the value of polarization is increased [69]. It was also observed that the trend of (*ω*) is a typical behavior for a polar dielectric as the value of (*ω*) increases up to a peak value at a certain temperature and then drops. This drop in can be ascribed to the intensified thermal vibrations of the dye molecules, which disturb the orderliness of the oriented dipoles [70,71]; a similar trend is applicable for the dependence of the dielectric loss (*ω*) with temperature. The dielectric properties of E143 food dye have screened a new OSC material with known energy alignment relaxation spectra and dielectric strength, which are directly related to the device stability and lifetime [72,73].

## 4. Conclusions

Based on the obtained results, E143 food dye (C_37_H_34_N_2_Na_2_O_10_S_3_) can be considered as an organic semiconductor material as confirmed by the measured value of room temperature of interband transition (E_int_ = 1.885 eV), DC conductivity (*σ_dc_* = 3 × 10^−6^ S/m), and activation energy (0.1 eV). This result has motivated our group to conduct future intensive studies on E143 dye (C_37_H_34_N_2_Na_2_O_10_S_3_) thin films for bandgap engineering and mobility optimization for OSC devices. The conduction mechanism has been explained on the basis of the OLPT model, which suggested the application of E143 food dye as a hole injection layer (HIL) for OLED applications. The dielectric properties were explicated on the basis of the Debye model for the frequency and temperature dependence for the typical semiconductor material. This study is significant as dielectric relaxations can strongly affect the performance and stability of OSC devices, such as transistors, capacitors, sensors, etc. Moreover, TGA measurements revealed that E143 food dye (C_37_H_34_N_2_Na_2_O_10_S_3_) exhibits excellent thermal stability over a wide range of climatic temperatures. E143 food dye (C_37_H_34_N_2_Na_2_O_10_S_3_) is recommended to be considered a sustainable OSC material for the future of green energy in hot countries like KSA.

## Figures and Tables

**Table 1:** XRD identification of Miller indices for E143 food dye (C_37_H_34_N_2_Na_2_O_10_S_3_) in powder form.

*hkl*	(obs.) 2θ°	(calc.) 2θ°	Δ(2θ°)
−110	12.2675	12.2742	−0.0067
11	14.8228	14.8149	0.0079
201	16.18	16.1646	0.0154
111	16.84	16.8394	0.0006
−102	17.3	17.314	−0.014
−310	18.0375	18.0471	−0.0096
2	19.0089	19.0397	−0.0308
211	19.82	19.7968	0.0232
−112	20.76	20.7521	0.0079
−412	22.54	22.512	0.028
500	23.4	23.4093	−0.0093
202	24.94	24.9403	−0.0003
−510	26.1	26.0874	0.0126
−320	26.86	26.8613	−0.0013
411	27.28	27.2892	−0.0092
−213	28.09	28.0867	0.0033
−122	28.8	28.7862	0.0138
321	30.74	30.7513	−0.0113
−711	31.76	31.8061	−0.0461
−700	33.04	32.9997	0.0403
421	33.89	33.9008	−0.0108

## Data Availability

The data that support the findings of this study are not publicly available due to privacy conditions.
